# Malvidin and Its Mono- and Di-Glucosides Forms: A Study of Combining Both In Vitro and Molecular Docking Studies Focused on Cholinesterase, Butyrylcholinesterase, COX-1 and COX-2 Activities

**DOI:** 10.3390/molecules28237872

**Published:** 2023-11-30

**Authors:** Paulina Strugała-Danak, Maciej Spiegel, Janina Gabrielska

**Affiliations:** 1 Department of Physics and Biophysics, Wrocław University of Environmental and Life Sciences, C. K. Norwida 25, 50-375 Wrocław, Poland; janina.gabrielska@upwr.edu.pl; 2 Department of Organic Chemistry and Pharmaceutical Technology, Wrocław Medical University, Borowska 211A, 50-556 Wrocław, Poland

**Keywords:** cyclooxygenase, cholinesterase, malvidin, molecular docking

## Abstract

Malvidin, one of the six most prominent anthocyanins found in various fruits and vegetables, may possess a wide range of health-promoting properties. The biological activity of malvidin and its glycosides is not entirely clear and has been relatively less frequently studied compared to other anthocyanins. Therefore, this study aimed to determine the relationship between the structural derivatives of malvidin and their anti-cholinergic and anti-inflammatory activity. The study selected malvidin (Mv) and its two sugar derivatives: malvidin 3-*O*-glucoside (Mv 3-glc) and malvidin 3,5-*O*-diglucoside (Mv 3,5-diglc). The anti-inflammatory activity was assessed by inhibiting the enzymes, specifically COX-1 and COX-2. Additionally, the inhibitory effects on cholinesterase activity, particularly acetylcholinesterase (AChE) and butyrylcholinesterase (BChE), were evaluated. Molecular modeling was also employed to examine and visualize the interactions between enzymes and anthocyanins. The results revealed that the highest inhibitory capacity at concentration 100 µM was demonstrated by Mv 3-glc in relation to AChE (26.3 ± 3.1%) and BChE (22.1 ± 3.0%), highlighting the crucial role of the glycoside substituent at the C3 position of the C ring in determining the inhibitory efficiency of these enzymes. In addition, the glycosylation of malvidin significantly reduced the anti-inflammatory activity of these derivatives compared to the aglycone form. The IC_50_ parameter demonstrates the following relationship for the COX-1 enzyme: Mv (12.45 ± 0.70 µM) < Mv 3-glc (74.78 ± 0.06 µM) < Mv 3,5-diglc (90.36 ± 1.92 µM). Similarly, for the COX-2 enzyme, we have: Mv (2.76 ± 0.16 µM) < Mv 3-glc (39.92 ± 3.02 µM) < Mv 3.5-diglc (66.45 ± 1.93 µM). All tested forms of malvidin exhibited higher activity towards COX-2 compared to COX-1, indicating their selectivity as inhibitors of COX-2. Theoretical calculations were capable of qualitatively replicating most of the noted patterns in the experimental data, explaining the impact of deprotonation and glycosylation on inhibitory activity. It can be suggested that anthocyanins, such as malvidins, could be valuable in the development of treatments for inflammatory conditions and Alzheimer’s disease and deserve further study.

## 1. Introduction

Alzheimer’s disease (AD) is viewed as a progressive, neurodegenerative pathology. Whereas the origins of AD are not fully explained, it is broadly accepted that it is closely linked to the impairment of cholinergic transmission, in which one of the neurotransmitter, namely acetylcholine, plays a vital role. Furthermore, cholinesterases, including acetylcholinesterase (AChE) and butyrylcholinesterases (BChE), are believed to be key enzymes which are able to hydrolyze acetylcholine [1,2]. As the result of this hydrolysis, there is a significant decrease in acetylcholine which is associated with the loss of basic forebrain cells, which, in turn, are key in order to determine the cognitive impairment of the brain [3]. The process of both enzymes’ inhibition, namely AChE and BChE, is the most effective and modern therapeutic approach in cognitive impairment [4,5].

Currently, various therapeutic strategies are being studied in order to develop an effective anti-Alzheimer’s drug. Yu et al. [6] recently identified microRNA-485-3p (miR-485-3p) as a biomarker and therapeutic target for AD. Their research primarily focused on miR-485-3p’s effects in cell-based in vitro systems, whereas in vivo physiological relevance was not viewed. Research conducted by Koh et al. [7] utilized the miR-485-3p marker in a transgenic mouse model of AD. They demonstrated the overexpression of miR-485-3p in the brain tissues of AD patients, and its antisense oligonucleotide (ASO), effectively reduced Aβ plaque buildup, tau pathology progression, neuroinflammation, and cognitive decline.

Furthermore, there is a vigorous exploration seeking non-toxic natural remedies for Alzheimer’s disease, where curcumin, known for its diverse biological properties [8,9,10], is under close investigation. The antioxidant potential of five mono-carbonyl curcumin analogs was assessed through in vitro tests and an in vivo mouse model focused on the hippocampus [11]. Among these analogs, two with methoxy and chloro-substitutions demonstrated strong DPPH and ABTS free radical scavenging capabilities in the in vitro assays. The authors also revealed a significant decrease in lipid peroxidation and enhanced activities of catalase, superoxide dismutase, and glutathione in the mouse hippocampus through marker analysis, highlighting their antioxidant and memory-enhancing properties. Khan et al. [12] conducted research on the alkaloids nuciferine and norcoclaurine extracted from N. nucifera seeds, highlighting their anti-Alzheimer’s and antioxidant properties in a diabetic rat model. The alkaloids notably restored AChE activity in both the blood and brains of the rats, along with the recovery of all antioxidant enzymes. The study showed that nuciferine and norcoclaurine substantially enhance memory and both could be effective phytomedicines against diabetes and Alzheimer’s disease (AD). Referring to the compounds from the group of flavonoids, isorhamnetin and quercetin derivatives, which were obtained in the process of isolating them from *Calendula officinalis* L., they are, among others, excellent examples of powerful AChE inhibitors [4,13]. The results of anticholinesterase activities of 24 polyphenolic compounds were interestingly presented in a brief report by Szwajgier [14]. The research was focused on a group of anthocyanins, flavones, flavanols, as well as dihydrochalcone phlorizin and prenylated chalcone xanthohumol. While analyzing the differences in properties in the molecular structure of those compounds, the author suggested that the inhibitory activity decreased in the presence of a 3-hydroxyl group; similarly, it was also stated that aglycons were more effective cholinesterase inhibitors than their corresponding glycosylated forms. Moreover, there are the other studies which proved the inhibitory activities of aqueous fruit mulberry extracts (*Morus* spp.) against AChE and BChE, which are not only rich in anthocyanins, but also in cyanidin, kuromanin, and keracyanin [15].

Oxidative stress and inflammation are both key causative factors for the onset of several diseases, including cancer, metabolic and cardiovascular disorders, and neurodegenerative diseases. Aging is responsible for heightening oxidative stress in the nervous system, leading to impaired nerve regeneration and function [16,17]. Furthermore, inflammation is detected in the human central nervous system and increased levels of inflammatory cytokines were observed in the cerebrospinal fluid of aging individuals [18]. 

The broad spectrum of potentially anti-inflammatory activity displayed by biologically active compounds can be tested by way of measuring the inhibition of the cyclooxygenase (COX-1, COX-2) activity [19,20]. These two enzymes take part in the synthesis of several mediators of inflammation, including prostaglandin PGE2, leukotriene B4, and thromboxane A2. As a result of the inhibition showed by anthocyanins, and which is generated by those enzymes, the synthesis of the mediators of inflammation, which are responsible for inhibiting leukocyte accumulation and adjusting the vascular system, decreases. Therefore, the overall effects of inflammation are minimalized. The above effects were proved by Fagundes et al. [21], who aimed to compare the anti-inflammatory effect of anthocyanin-rich extract from blueberries (with malvidin-3-galactoside and petunidin-3-arabinoside) with 5-aminosalicylic acid in the TNBS-induced colitis model. It was showed that the anthocyanin extract at a concentration 30 times lower than 5-aminosalicylic acid had higher effectiveness in counteracting intestinal inflammation. Furthermore, it was Seeram et al. [22] who proved, using numerous anthocyanin extracts from berries and cherries, that anthocyanins from raspberries and sweet cherries demonstrated 45% and 47% COX-1 and COX-2 inhibitory activities respectively, referring to assays at 125 µg/mL. Additionally, studies showed that aglycone cyanidin has higher inhibitory activity in comparison to its glycosides, and that COX inhibitory activities increased with a decreasing number of sugar residues, which were attached to the cyanidin moiety. 

Malvidin, named 3,5-dimethoxy-3,4,5,7-tetrahydroxyflavylium acid anion, is a type of anthocyanidin cation with a chemical structure similar to delphinidin but with methyl groups attached to positions 3 and 5. In its natural state, it is predominantly encountered in its glycosylated form, with a sugar moiety attached at position 3 on the C-ring. Several studies, both in vitro and in vivo, indicate that these molecules have the potential to mitigate the onset and progression of various disease pathologies, particularly those associated with oxidative stress-related pathogenesis [16]. Previously, we conducted an experiment that proved that Mv and its two glucosides (Mv 3-glc > Mv 3,5-diglc) have a tendency to exhibit in vitro (AAPH^●^ and DPPH^●^ assays) antioxidant activities against liposome membrane in the order illustrated here: Mv > Mv 3-glc > Mv 3,5-diglc. Moreover, we observed that this activity is in line with interaction with this membrane [23]. Despite the fact that the anti-cholinesterase and anti-inflammatory properties of anthocyanins were carefully investigated, there is limited access to information on the effects of such glycosylation on the mechanisms developed by anthocyanins in order to manage/prevent disease conditions. In the experiment that we are describing in this paper, we endeavor to demonstrate the ability of these anthocyanins to inhibit the cholinesterase enzymes and the pro-inflammatory cyclooxygenases COX-1 and COX-2. 

As a result, this study is intended to compare the anticholinergic activity and cyclooxygenase (COX-1 and COX-2) inhibitory (as potential anti-inflammatory) properties of Mv and its mono- and di-glucosides. It is important to underline that in this study, for the first time, calculations were carried out with the usage of a computational chemistry approach, which is the molecular docking method, in order to demonstrate the relationship between the anticholinesterase and anti-inflammatory effectiveness of Mv-s and their molecular structure with regard to their interconnections at the atomic level. We hope that the outcome obtained here as a result of these complementary studies will be beneficial in order to indicate what are the potential methods of application for Mv and its mono- and di-glucosides in order to save human lives and treat the early stages of neurodegenerative and anti-inflammatory diseases. 

## 2. Results and Discussion

### 2.1. Acetylcholinesterase and Buthyrylcholinesterase Inhibition

Anti-cholinergic activity was assessed by inhibiting the enzymes acetylcholinesterase and butyrylcholinesterase specifically. Inhibiting both enzymes is the most effective therapeutic approach for restoring the normal functions of the cholinergic system. Therefore, effective pharmaceuticals are being sought, in particular natural inhibitors of these enzymes. The results of our studies, which were focused on the use of Mv and its two derivatives, as well as the drug neostigmine, are summarized in Table 1, which contains the percentages of AChE and BChE inhibition for 100 µM anthocyanin concentration. There was no dependence on the concentration of anthocyanins within the range 100–200 µM. The highest inhibitory capacity in relation to both enzymes was demonstrated by Mv 3-glc −26.31 ± 3.06% and 22.07 ± 3.00%, in relation to AChE and BChE, respectively. Both Mv and Mv 3,5-diglc were less potent enzyme inhibitors compared to Mv 3-glc. On the one hand, referring to the differences in molecular structure between Mv 3-gluc and Mv, one may suggest the importance of the glycoside substituent in the C3 position of the C ring of the molecule on the inhibitory efficiency of the enzymes of this mono-glycoside. On the other hand, a reduction in this effectiveness occurs after replacing the hydroxyl group at the C5 position with another, second glucoside molecule. In order to reveal fully the relationship between the structure of anthocyanins and the inhibitory efficacy of AChE and BChE enzymes, further research is required. The neostigmine drug used in the treatment of old age disease, AD, showed 50% inhibition efficiency in relation to AChE and BChE at concentration rates of 0.17 ± 0.01 µM and 20.28 ± 1.70 µM, respectively. By relating the inhibition values expressed in % in relation to the results caused by Mv 3-glc, it can be roughly stated that the concentration of this anthocyanin should be increased several times, e.g., 5–10 times in relation to BChE, in order to have an effect similar to neostigmine. Bearing in mind the fact that anthocyanins are natural, non-toxic substances found in red and purple fruits and vegetables, the process of increasing the concentration of these substances several times is possible through supplementation with nutraceuticals. However, there is a serious problem with their low bioavailability and molecular instability under physiological conditions. The core of this problem lies in finding effective solutions proposed by further research, which should be focused on, e.g., the design of encapsulated anthocyanins or extracts with a high content of the compounds required [24,25,26] 

Literature data shows potential AChE inhibitory activity in over 300 natural compounds, including alkaloids (53%), monoterpenes (10%), coumarins (7%), triterpenes (6.5%), flavonoids (5%), simple phenols (5%) and others [13,27]. In the case of compounds obtained from the group of flavonoids, isorhamnetin and quercetin derivatives isolated from *Calendula officinalis* L. are other examples of potential AChE inhibitors [13]. A study conducted by Yusuf et al. [28], which was focused on the inhibition of AChE and BChE by 12 colored carrot varieties, demonstrated the effectiveness of micro purple carrot (MPC) and normal purple carrot (NPC) in inhibiting these enzymes. Whereas IC_50_ values in relation to AChE for purple carrot MPC and NPC were 10.14 mg/mL (i.e., 10140 µg/mL) and 18.96 mg/mL, respectively, in relation to the BChE enzyme they were lower and comparable to each other, at 7.83 mg/mL and 7.85 mg/mL, respectively. The authors of this study also determined the content of anthocyanins, phenolic compounds, and carotenoids in the most active MCP and NCP variants. It was observed that the content of these active compounds was lower in the MPC (namely 53.05 mg/100 g dm, 128.34 mg/100 g dm and 18.91 mg/100 g dm, respectively) than in the NPC, in which these compounds were at a higher level (namely 69.62 mg/100 g dm, 253.3 mg/100 g dm, and 19.07 mg/100 g dm). While comparing these values, it is clear that they did not allow the authors of this study to indicate/explain why micro purple carrot has a higher inhibitory activity in relation to the AChE and BChE enzymes than normal purple carrot, which is richer in these biologically active substances. It is also noticed, by referring to the IC_50_ values determined by Yusuf et al. [28] that the percentage of inhibition obtained in this study, determined for the concentration of Mv-s—100 µM, was about 78 to 190 times higher. 

The antioxidant and free radical scavenging and aging-related enzymes, such as AChE and BChE, showed certain properties demonstrated in the study, which proved they are potent compounds, as well as being able to inhibit/delay free radical aging processes observed in the organism. Malvidin and its glycosides have been reported to positively impact neurodegenerative disease [16,29]. Lin et al. [30] demonstrated that preincubation with malvidin increased CAT activity and GSH concentration after hypoxia treatment, and cells also showed higher SOD activities. Zhao et al. [31] in a study on a murine microglial cell line revealed that malvidin prevented mitochondrial dysfunction, reduced ROS accumulation and lipid peroxidation, and increased antioxidant enzyme activity in the cerebrum. Giliani et al. [32] confirmed the neuroprotective effects of orally administered malvidin, regulating antioxidant levels and neuroinflammation in rats exposed to AlCl_3_. In summary, research clearly supports that malvidin possesses antioxidant activity by inhibiting acetylcholinesterase and managing oxidative stress in neuronal cells.

### 2.2. Anti-Inflammatory Activity—COX Inhibition 

The potential anti-inflammatory activity of Mv, Mv 3-glc and Mv 3,5-diglc was proved on the basis of inhibition of the COX activity. Anthocyanins/flavonoids, also known as nature’s tender drugs, possess various pharmacological activities, including antioxidant and anti-inflammatory activities. These compounds slow down enzyme activities of arachidonic acid metabolizing enzymes, such as phospholipase PLA2, cyclooxygenase, lipoxygenase and others [33,34]; the inhibition of these enzymes, which is caused by anthocyanins, including cyclooxygenases, is definitely one of the important cellular mechanism of anti-inflammation [35]. The results of our studies, summarized in Table 2, contain the concentration values of IC_50_ for Mv and its glycoside derivatives, which inhibit the activity of COX-1 and COX-2 by 50%. The data presented in Table 2 indicate that Mv had the greatest ability to inhibit both COX-1 and COX-2. The glycosylation of malvidin significantly reduced the anti-inflammatory activity of these derivatives compared to Mv. This reduction was within the range of about 6–7 times in relation to COX-1, and within the range of about 14.5–24 times in the case of COX-2. 

In Table 2 the anthocyanin selectivity index, i.e., the COX-2/COX-1 ratio, is also included. In our research, we obtained a COX-2/COX-1 selectivity ratio of 0.22, 0.53, and 0.83 for Mv, Mv 3-glc, and Mv 3,5-diglc, respectively. This indicates that the anthocyanins exhibit approximately 4.5 to 1.4 times higher activity toward COX-2 than COX-1. Medical anti-inflammatory drugs, including NSAIDs, can cause side effects, e.g., gastrointestinal bleeding, cancer promotion, etc. [36]. In our opinion, it is highly likely that anthocyanins/flavonoids have multiple cellular mechanisms acting on multiple sites of cellular machinery; among them, one is responsible for anti-inflammation. which seems to be caused by an effect on eicosanoid generating enzymes (COX1 and COX2 and others. like 5-, 12-LOX, PLA2). It is possible that they can also, similarly to steroidal anti-inflammatory drugs, stimulate protein kinases PKC, PTK and MAPK and down-regulation of the expression of iNOS, TNF-α, IL-1β [37]. Demonstration of such possible modes of action of anthocyanins should be proved in further studies.

Referring to the literature data, which is concentrated on studies of malvidin and its sugar derivatives, and extracts rich in these anthocyanins, it may be concluded that they are connected with numerous anti-inflammatory mechanisms, a fact which was proved in various in vitro models. In the article prepared by Huang et al. [38], the effect of the malvidin-3-glucoside and malvidin-3-galactoside obtained from blueberry *Vaccinium ashei* on inflammatory response in endothelial cells was tested. Particularly, the authors discussed indicated the mechanism of anti-inflammatory activity of malvidin glucosides as a way of restraining tumor necrosis factor-alpha (TNF-α), and by nuclear factor-kappa B (NF-κB). Generally, in these processes, malvidin-3-glucoside exerted a more powerful effect than malvidin-3-galactoside. Fagundes et al. [21] studied the potential of anthocyanidin malvidin, with an intention to protect against and help in peptic ulcer treatment. Expression levels of oxidative and inflammatory genes in the mouse gut in the presence of a 5 mg· kg^−1^ dose were determined in order to investigate the mechanism of malvidin activity. Malvidin, thus. was proved to prevent gastric and duodenal ulcers. As a result, significant anti-inflammatory and anti-oxidative effects on the gastrointestinal tract connected with gene expression modulation and, similarly, an increase in endogenous defense mechanisms were observed. Three anthocyanin di-glucosides from *Syzygium cumini* pulp were isolated in the study conducted by Abdin et al. [39], including delphinidin 3,5-diglucoside, petunidin 3,5-diglucoside, and malvidin 3,5-diglucoside (MDG), with the malvidin derivative isolated at the highest yield. Moreover, MDG showed significant progress in inhibiting nitric oxide release and pro-inflammatory mouse cytokines, such as IL-1 beta, TNF-alpha and IL-6 in lipopolysaccharide-induced RAW264.7 macrophages. This experiment allowed us to better understand the structure–activity correlations of anthocyanin di-glucosides. The aim of yet another study was to examine the impact of malvidin on inflammatory responses and oxidative stress in peripheral blood mononuclear cells (PBMCs), which was generated by lipopolysaccharide (LPS). The most up-to-date studies proved that LPS significantly increased the (gene/cytokine expression) cytokine expression of IL-6, TNF-alpha, IL-1 beta, and COX-2 mRNA, and protein release from PBMCs, 22 h after treatment, whereas the expression of these cytokines (IL-6, TNF-alpha, IL-1 beta) and COX-2 mRNA were induced by LPS, secretion of protein in PBMC, and was much lower as a consequence of pretreatment of malvidin [40]. Finally, it is important to underline that there are also studies focusing on extracts rich in anthocyanins, inter alia malvidins and its sugar derivatives, as well as sugar cyanidin derivatives, which in vitro show their potential significance as drugs and substances which are in support of the treatment of inflammatory processes [41,42,43].

**Table 2:** Values of IC_50_ for malvidin (Mv), malvidin 3-*O*-glucoside (Mv 3-glc) and malvidin 3,5-*O*-diglucoside (Mv 3,5-diglc) of cyclooxygenase-1 (COX-1) and cyclooxygenase-2 (COX-2) and, for indomethacin cited as positive control, selectivity index COX-1/COX-2 is included. Different letters (^a–d^) in the same row indicate significant differences (*p* < 0.05).

Compound	IC_50_ (µM)		
	COX-1	COX-2	Selectivity Index COX-2/COX-1
Mv	12.45 ± 0.70 ^d^	2.76 ± 0.16 ^d^	0.22
Mv 3-glc	74.78 ± 0.06 ^b^	39.92 ± 3.02 ^b^	0.53
Mv 3,5-diglc	90.36 ± 1.92 ^a^	66.45 ± 1.93 ^a^	0.83
Indomethacin *	18.32 ± 0.40 ^c^	15.22 ± 1.36 ^c^	0.83

* Strugała et al. [44].

### 2.3. Computational Outcomes

With the exception of the cationic structure, which is virtually absent at physiological pH, we conducted docking studies on all other deprotonated species that exist in significant populations. Subsequently, these structures underwent molecular dynamics simulations. We were able to qualitatively reproduce the experimental data to a large extent. Throughout the 10 ns of MD simulations, the RMSD of the complexes under examination remained stable, indicating good structural stability. The RMSF demonstrated similar behaviour, with only marginal deviations associated with the terminal regions, while the remaining ones exhibited highly superimposable behaviour. Additionally, the small extent of the fluctuations in Rg provided assurance of the stability of the formed systems. Supplementary Materials provide detailed information on these topics. 

#### 2.3.1. Inhibitory Activity towards AChE and BChE

**Figure 1:**
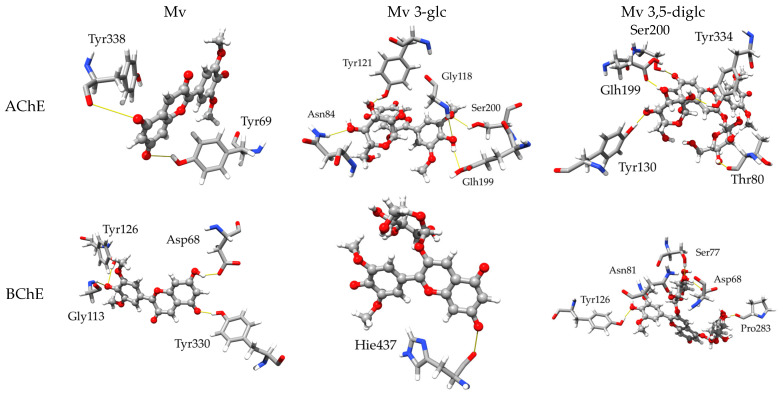
Post-MD 3D representations of the finest docked positions of Mv, Mv 3-glc and Mv 3,5-diglc species interacting with amino acids within the AChE and BChE binding pockets.

In the theoretical investigation of AChE inhibition, we were able to replicate the experimental findings on malvidin. Selectivity towards AChE is evident in both the experimental and theoretical approaches, indicated by a greater % of inhibition or lower inhibition constant, respectively. The interaction with the given enzyme appears to be modulated by hydrogen bonds originating from the Tyr338 and Tyr69 amino acids present in the binding pocket. This is different from BChE, where Tyr126, Asp68, Gly113 and Tyr330 participate in binding the ligand. See Figure 1 below.

For monoglucoside, experimental results indicate more effective inhibition towards AChE, whereas theoretical studies demonstrate lower *K*_i_ values for binding towards BChE (see Table 3 below). Nevertheless, selectivity varies only slightly in either case. It is important to note the possibility of the ligand interacting outside the binding pocket, such as via allosteric modulation. These factors could impact the behaviour in this case. This may be expected since, in comparison to malvidin, its glucoside interacts effectively with a greater number of amino acids, thus amplifying the stabilization effect through hydrogen bonding interactions.

The experimental data confirm that the diglucoside is preferentially inhibitory towards BChE. However, the computational study suggests significant divergence in inhibition outcomes between AChE and BChE, compared to the experimental data. Similar to monoglucoside, Glh199 and Ser200 participate in this interaction in AChE, while Asp68 and Tyr126 are common between malvidin and its diglucoside in BChE.

Deprotonation appears to result in improved inhibition of AChE by Mv, and even to a greater extent, by diglucoside. However, the effect is less pronounced in the case of monoglucoside. The inhibition of BChE displays a similar deprotonation trend to that of AChE. The addition of a sugar moiety through glycosylation leads to a reduction in inhibitory potential. This effect is more pronounced when two residues are added as opposed to a single glucose. The variations can reach up to three orders of magnitude. However, in the case of BChE, the impact of glycosylation is less noticeable as the diglucoside binds better than the aglycone.

#### 2.3.2. Inhibitory Activity towards COX-1 and COX-2

The assays showed a general preference for COX-1, but computational data only found this behavior for diglucoside. The differences in inhibition constants vary by up to three orders of magnitude for monoglucoside, whereas for aglycone and diglucoside this is only a difference of a single decimal place (see Table 4 below). However, the previously presented IC_50_ is high, which may lead to fluctuation in assessment and make it more prone to errors.

The process of deprotonation seems to improve the inhibitory activity towards COX-1 to some extent, while the reverse effect is observed for COX-2. Therefore, the hydrophobic nature of interactions appears to be of the utmost importance. 

**Figure 2:**
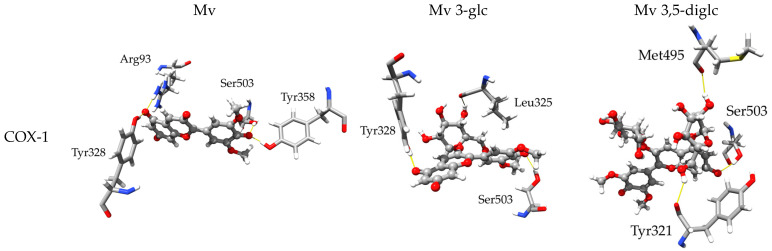

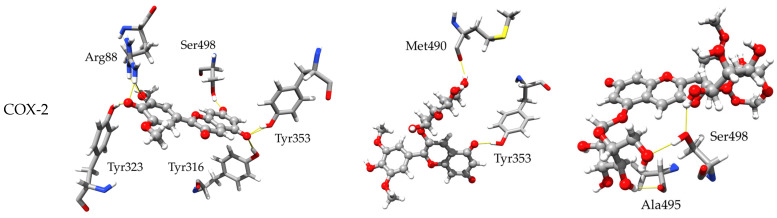
Post-MD 3D representations of the finest docked positions of Mv, Mv 3-glc and Mv 3,5-diglc species interacting with amino acids within the COX-1 and COX-2 binding pockets.

Moreover, it is apparent that glycosylation causes a significant increase in inhibition constants, indicating that a higher concentration is required to observe the inhibition process. Certainly, it is evident that glucosides can either shield the formation of stronger, more desirable bonding that retains the ligand in a pocket (see Figure 2 below), or block its further propagation deeper into the binding site, thus indirectly reducing the observed inhibition.

## 3. Materials and Methods

### 3.1. Materials

**Figure 3:**
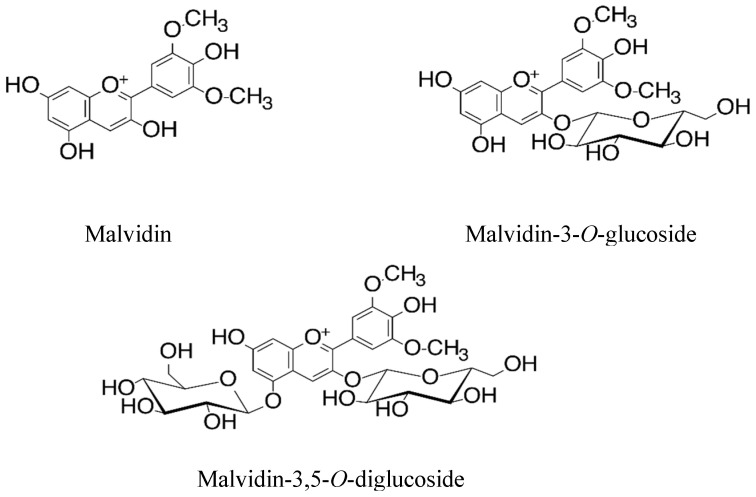
Chemical structures of the malvidin group.

5,5′-Dithiobis(2-nitrobenzoic acid), (DTNB), acetylthiocholine iodide, butyryl-thiocholine iodide, acetylcholinesterase from Electrophorus electricus, type VI-S (aChE), Butyrylcholinesterase from equine serum (BChE), neostigmine bromide, and sodium dodecyl sulfate (SDS) were purchased from Merck kGaA, Darmstadt, Germany. COX-1 and COX-2 colorimetric inhibitor screening assay kits (Catalog No. 701050) were purchased from Cayman Chemical, Ann Arbor, MI, USA. Malvidin (purity ≥ 97%), malvidin 3-*O*-glucoside (purity ≥ 95%) and malvidin 3,5-*O*-diglucoside (purity ≥ 95%), (Figure 3) were purchased from Extrasynthese (Genay, France), and all other chemicals were of analytical grade.

### 3.2. Acetylcholinesterase and Butyrylcholinesterase Inhibition Assay

Referring to both the Ellman [45] and Jin et al. [46] methods, we identified acetylcholinesterase/butyrylcholinesterase activity, implementing minor modifications. In brief, the assay was carried out on a 96-well plate, with each well containing 140 µL 0.1 M phosphate buffer (pH 8.0), 10 µL of stock solution of Mv, or Mv 3-glc or Mv 3,5-diglc, 10 µL AChE/BChE (1 units/mL). The plate was incubated for 10 min at 25 °C. Subsequently, 10 μL of a 10 mM DTNB was added to the reaction mixture. In the following step, the reaction was initiated by adding 10 μL of a 14 mM acetylthiocholine iodide/butyryl-thiocholine iodide. The plate was shaken for one minute and finally 20 µL of 5% SDS was added in order to stop the reaction. Control wells, which contained the same composition except for the studied compounds (10 µL, 70% ethanol), were included. Absorbance at λ = 417 nm was recorded using a plate reader (EPOCH, Bio Tech, Dover, MA, USA) after 10 min incubation. All of the reactions were performed in five repetitions. The percent of AChE/BChE inhibition was calculated using the following formula:

(1) $$\%Inhibition=\frac{A_{c}-A_{s}}{A_{c}}\cdot 100\%$$

where: A_s_ refers to the absorbance of the sample containing Mvs and Ac refers to the absorbance of the control sample. The studies of AChE and BChE inhibition were also performed depending on the concentration of the drug, neostigmine, as a positive control.

### 3.3. Cyclooxygenase (COX) Inhibition Assay

We determined the inhibition of both COX-1 and COX-2 activity using a colorimetric inhibitor screening assay kit (Catalog No. 701050), following the manufacturer’s instructions and as was previously explained by Jang and Pezzuto [47] and Strugała et al. [48]. The reaction mixtures were prepared in 0.1 M Tris–HCl buffer, pH 7.4.0 containing 10 μL hemin, 10 μL COX-1 (ovine) or COX-2 (human recombinant) and 10 μL Mv, Mv 3-glc Mv 3,5-diglc at final concentrations: 0.9–46 μM. Firstly, the plate was carefully shaken for a few seconds and incubated for 5 min at 25 °C. Secondly, 20 μL of colorimetric substrate solution was added to each plate’s well. It was the addition of 20 μL of arachidonic acid (final concentration in reaction mixture 100 µM) which initiated the reaction. Thirdly, the plate was shaken again for a few seconds and incubated for two min at 25 °C. The cyclooxygenase activity was assayed colorimetrically in a way of monitoring the appearance of oxidized TMPD at λ = 590 nm using a plate reader (EPOCH, Bio Tech). The percentage of COX inhibition was calculated using the Equation (1):

The IC_50_ parameter was calculated referring to plots showing the relation between percentage of COX inhibition and concentration of the compounds.

### 3.4. Computational Studies

Molecular docking was performed using AutoDock Vina 1.2.0 [49,50] on all structures that were present at physiological pH and had been established in the previous publication [23]. To obtain representative polyphenol:protein complexes, the existing bound ligand was used in the docking process. 

Enzyme crystal structures were obtained from the RSCB PDB database and consisted of human COX–1 (PDB ID: 6Y3C, res. 3.36 Å) [51] and human COX-2 bound with meclofenamic acid (PDB ID: 5IKQ, res. 2.41 Å) [52], the structure of human acetylcholinesterase (AChE) bound with donepezil (PDB ID: 6O4W, resolution [53] 2.35 Å, and butyrylcholinesterase (AChE) bound with profenamine (PDB ID: 5K5E, resolution 2.80 Å [54]. They were chosen for analysis due to the presence of inhibitors, fair resolution, unmutated *H*. *sapiens* source of origin, and the absence of any missing loops, except for terminal segments.

To ensure complete residue coverage within 5 Å of the inhibitor’s mass center, we employed a docking box. We redocked the initial ligands, and the process succeeded. Unfortunately, we could not find a suitable enzyme inhibitor complex for COX-1, so we overlaid it with COX-2, which shares a binding pocket [51]. Next, we executed the docking process in the relevant box for the meclofenamic acid binding site. As the energy differences and positions of the overall results were not significant between docking outcomes, we conducted molecular dynamics simulations with the best one. Prior to that, we used the corresponding ΔG_binding_ values [55] to determine the inhibition constant *K_i_* [M] by following the equation:

(2) $$K_{i}=exp\left(\frac{-{∆G}_{binding}}{RT}\right)$$

Before commencing the molecular dynamics, all ligands were parametrized. Gas-phase geometry optimization was carried out with HF/6-31G*. The electrostatic potential was fitted according to the Merz–Singh–Kollman scheme [56] to determine atomic charges, using the RESP procedure. The GAFF2 force field [57] was utilized to extrapolate the parameters. To generate the necessary files, the Antechamber and parmchk modules of Amber16 were utilized [58].

Subsequently, a refinement process was performed on the enzymes to eliminate any superfluous molecules, including crystallographic water and solvents. The protonation states of titratable residues at pH 7.4 were then estimated, using the PDB2PQR server [59,60] and the PropKa algorithm [61]. Each complex was solvated with a TIP3P water box and, in order to maintain system electroneutrality, either sodium or chloride counterions were added. After conducting initial system optimization and heating to 310 K within the NVT and NPT ensembles, 10 ns production simulations were performed for each complex at the equivalent temperature and pressure. The simulations utilized a time constant of 2.0 ps and employed the SHAKE algorithm [62] and Particle Mesh Ewald [63], which made way for an integration step of 2 fs. Further, a cut-off radius of 1.2 nm was utilized. The representative structures were identified from the most populous cluster of the molecular dynamics results.

All simulations were performed using the Gromacs software 2022.2 package [64], whilst the UCSF Chimera software 1.17.3 [65] was employed to analyze the interactions.

### 3.5. Statistical Analysis

Data are shown as mean values ± standard deviation (SD). *p* values < 0.05 were considered statistically significant. Statistical analysis was performed using the program Statistica 12.0 (StatSoft, Kraków, Poland).

## 4. Conclusions

Our in vitro study demonstrates that malvidin, as well as its glucosides, exert on COX-1, COX-2 and AChE, BChE an enzyme inhibitory effect. In addition, it was shown that the presence of sugar residues affected the anti-inflammatory and anti-cholinergic activity properties. This demonstrates that the glycosylation of malvidin substantially diminished its anti-inflammatory activity. The computational protocol led us establish the role of protonation and deprotonation in the inhibitory activity, as well giving insights into the amino acids comprising the given behaviour. 

To sum up, despite significant progress being made in the search for natural products with antioxidant properties, understanding of the structure–activity relationships (SARs) among anthocyanins and their effects on enzyme inhibitors remains incomplete, which is why it is necessary to fully elucidate their relationship. 

## Figures and Tables

**Table 1:** Cholinesterase inhibitory activity (against AChE and BChE) of the malvidin (Mv), malvidin 3-*O*-glucoside (Mv 3-glc) and malvidin 3,5-*O*-diglucoside (Mv 3,5-diglc). Concentration of Mv, Mv 3-glc and Mv 3,5-diglc was 100 µM. Neostigmine concentration for AChE inhibition was 0.17 µM, and, for BChE inhibition, it was 20.28 µM. Different letters (^a–c^) in the same row indicate significant differences (*p* < 0.05).

Compound	Inhibition of AChE (%)	Inhibition of BChE (%)
Mv	19.5 ± 2.3 ^b^	10.2 ± 2.2 ^b^
Mv 3-glc	26.3 ± 3.1 ^a^	22.1 ± 3.0 ^a^
Mv 3,5-diglc	11.9 ± 4.5 ^c^	13.4 ± 2.4 ^b^
Neostigmine	50.0 ± 1.2	50.0 ± 4.2

**Table 3:** Theoretically estimated inhibition constant (in M) of each species with a non-negligible population towards the AChE and BChE enzymes.

Compound	^f^ *M*	AChE	BChE
Mv	39.56%	2.58 × 10^−7^	1.52 × 10^−6^
Mv^−^	52.15%	1.89 × 10^−7^	1.19 × 10^−6^
Mv^2−^	8.08%	1.16 × 10^−7^	6.41 × 10^−7^
Mv^3−^	0.13%	8.04 × 10^−8^	6.42 × 10^−7^
	***K_i-overall_***	***2.10*** ***× 10^−7^***	***1.28*** ***× 10^−6^***
Mv 3-glc	3.49%	2.77 × 10^−6^	5.39 × 10^−7^
Mv 3-glc^−^	78.18%	2.42 × 10^−6^	5.99 × 10^−7^
Mv 3-glc^2−^	18.33%	2.88 × 10^−6^	4.12 × 10^−7^
	***K_i-overall_***	***2.52*** ***× 10^−6^***	***5.63*** ***× 10^−7^***
Mv 3,5-diglc	39.19%	4.51 × 10^−3^	3.70 × 10^−7^
Mv 3,5-diglc^–^	60.70%	1.63 × 10^−4^	8.99 × 10^−8^
	***K_i-overall_***	***1.87*** ***× 10^−3^***	***1.99*** ***× 10^−7^***

**Table 4:** Theoretically estimated inhibition constant (in M) of each species with a non-negligible population towards the COX-1 and COX-2 enzymes.

Compound	^f^ *M*	COX-1	COX-2
Mv	39.56%	9.08 × 10^−4^	5.42 × 10^−5^
Mv^−^	52.15%	8.18 × 10^−4^	6.68 × 10^−5^
Mv^2−^	8.08%	6.41 × 10^−4^	4.85 × 10^−5^
Mv^3−^	0.13%	5.46 × 10^−4^	3.92 × 10^−5^
	***K_i-overall_***	***8.38*** ***× 10^−4^***	***6.02 × 10^−5^***
Mv 3-glc	3.49%	3.43 × 10^2^	2.41 × 10^−3^
Mv 3-glc^−^	78.18%	2.89 × 10^2^	1.15 × 10^−1^
Mv 3-glc^2−^	18.33%	2.64 × 10^2^	2.90 × 10^−1^
	***K_i-overall_***	***2.87 × 10^2^***	***1.43*** ***× 10^−1^***
Mv 3,5-diglc	39.19%	1.34 × 10^11^	1.98 × 10^9^
Mv 3,5-diglc^−^	60.70%	1.12 × 10^11^	3.20 × 10^12^
	***K_i-overall_***	***1.21*** ***× 10^11^***	***1.95*** ***× 10^12^***

## Data Availability

Data is contained within the article.

## Supplementary Materials

The following supporting information can be downloaded at: https://www.mdpi.com/article/10.3390/molecules28237872/s1.
