# Supplementary material for: Malvidin and Its Mono- and Di-Glucosides Forms: A Study of Combining Both In Vitro and Molecular Docking Studies Focused on Cholinesterase, Butyrylcholinesterase, COX-1 and COX-2 Activities
Source: Molecules. 2023 Nov 30;28(23):7872. doi: 10.3390/molecules28237872 (PMC10708353; doi:10.3390/molecules28237872)
Supplement: Supplementary file 1 [file molecules-28-07872-s001.zip › molecules-2645833-supplementary.pdf]

SUPPLEMENTARY MATERIALS

Malvidin and its mono- and di-glucosides forms: a study of combining both *in vitro* and molecular docking studies focused on cholinesterase, butyrylcholinesterase, COX-1 and COX-2 activities

Paulina Strugała-Danak[1,*], Maciej Spiegel[2,*] and Janina Gabrielska[1]

Department of Physics and Biophysics, Wrocław University of Environmental and Life Sciences, C. K. Norwida 25, 50-375 Wrocław, Poland; janina.gabrielska@upwr.edu.pl

2 Department of Organic Chemistry and Pharmaceutical Technology, Wrocław Medical University, Borowska 211A, 50-556 Wrocław, Poland; maciej.spiegel@umw.edu.pl

* Correspondence: paulina.strugala@upwr.edu.pl ; Tel.: +48-71-320-5295, maciej.spiegel@umw.edu.pl

Figure S1. Radius of gyration, RMSD and RMSF calculated for the backbone atoms of the complexes with COX-1.

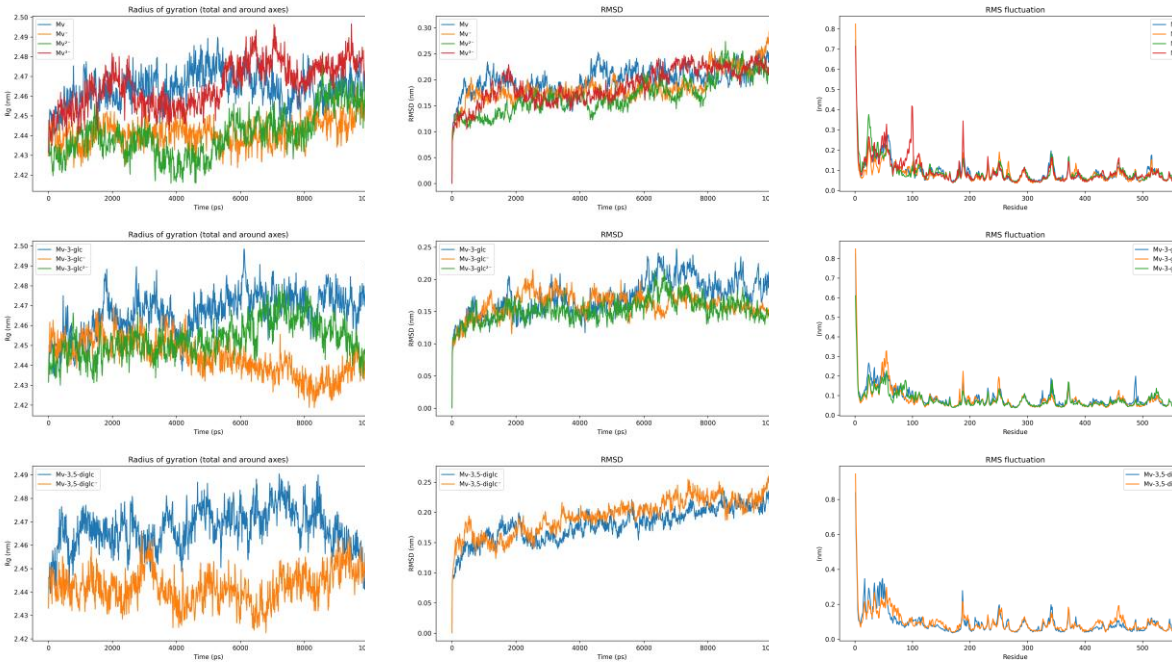

Figure S2. Radius of gyration, RMSD and RMSF calculated for the backbone atoms of the complexes with COX-2.

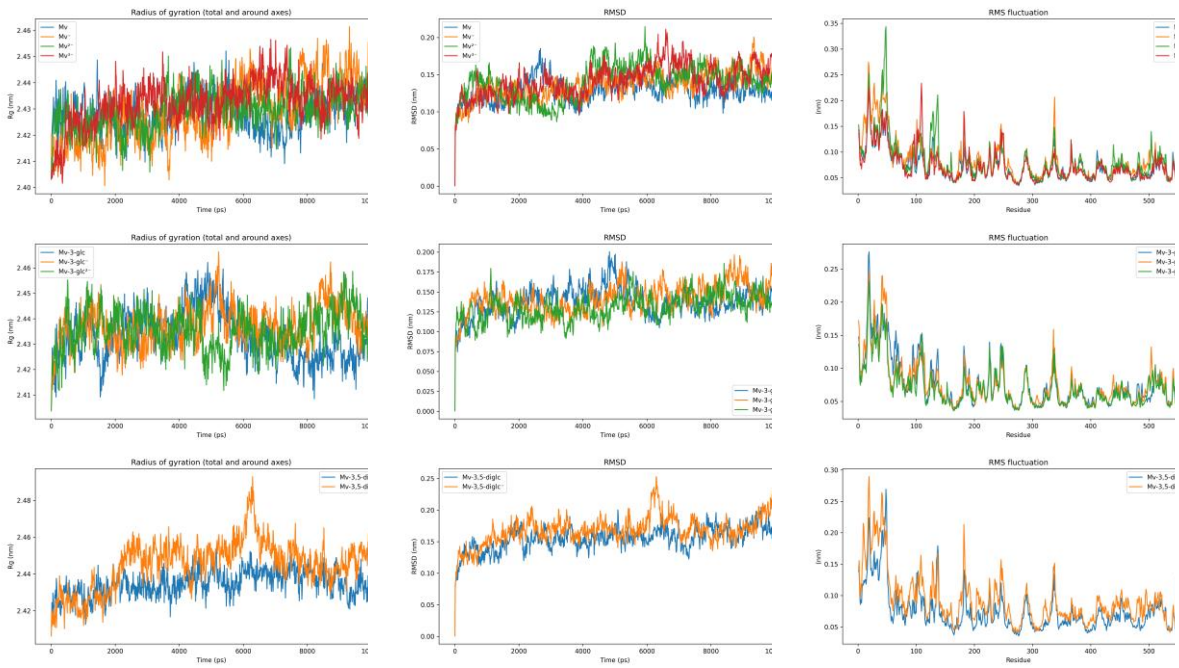

Figure S3. Radius of gyration, RMSD and RMSF calculated for the backbone atoms of the complexes with AChE.

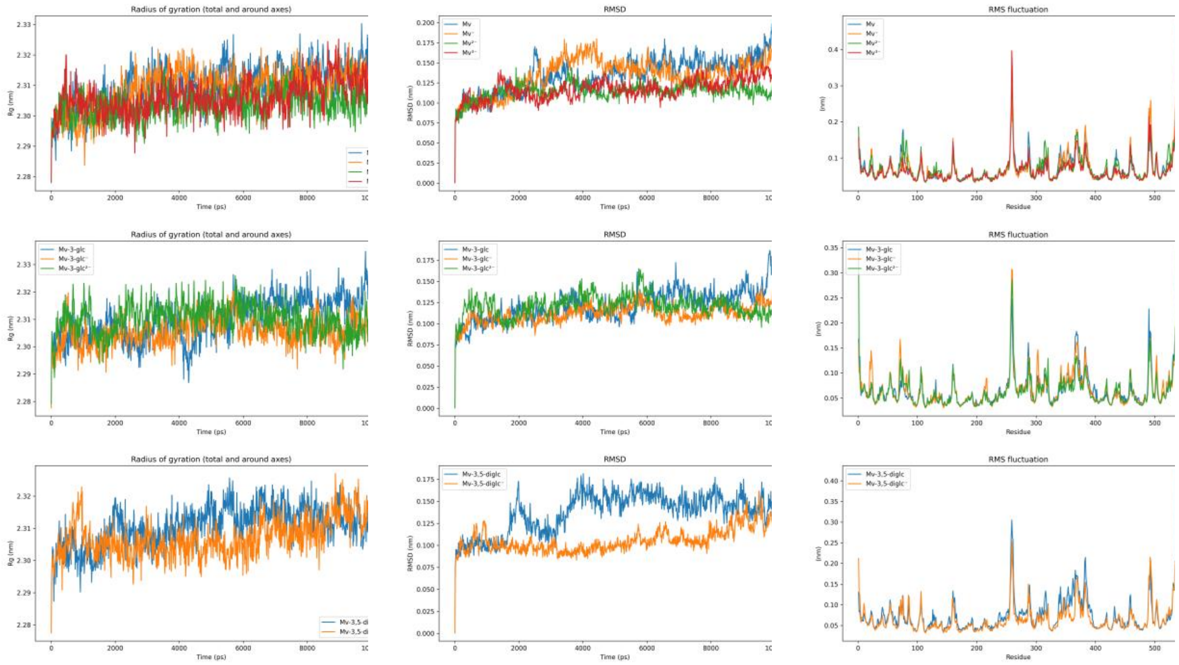

Figure S4. Radius of gyration, RMSD and RMSF calculated for the backbone atoms of the complexes with BChE.

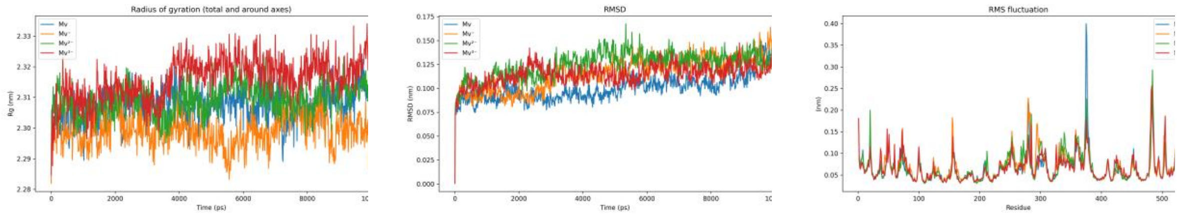

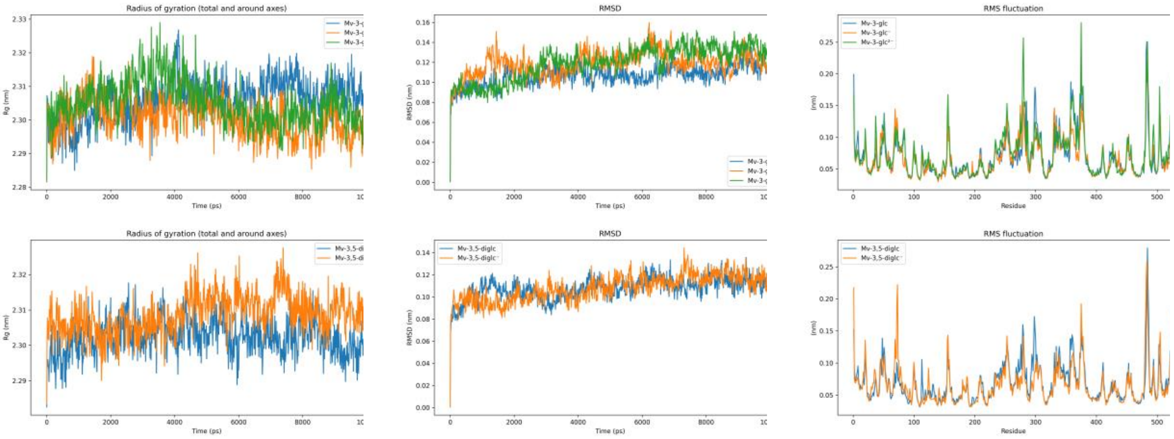

Radius of gyration (total and around axes)
Rg (nm)
Time (ps)
RMSD
RMSD (nm)
Time (ps)
RMS fluctuation
Mv-3-glc
Mv-3-glc^{-}
Mv-3-glc^{2-}
(nm)
Residue
Radius of gyration (total and around axes)
Rg (nm)
Time (ps)
RMSD
Mv-3,5-diglc
Mv-3,5-diglc^{-}
RMSD (nm)
Time (ps)
RMS fluctuation
Mv-3,5-diglc
Mv-3,5-diglc^{-}
(nm)
Residue
